# A Five-Year Retrospective Analysis of Diagnostic and Treatment Data of Flexor Sheath Infections: Can We Accurately Predict the Presence and Severity of Infection Prior to Surgical Washout?

**DOI:** 10.7759/cureus.19715

**Published:** 2021-11-18

**Authors:** Joseph Muscat, Robert Manton, Rowaa Ahmed, Oscar Johnson, Hyder Ridha, Patrick Goon

**Affiliations:** 1 Trauma and Orthopaedics, East and North Hertfordshire NHS Trust, Stevenage, GBR; 2 Plastic Surgery, Addenbrooke's Hospital, Cambridge University Hospitals NHS Foundation Trust, Cambridge, GBR; 3 Plastic Surgery, Leeds General Infirmary, Leeds, GBR; 4 Orthopaedics and Trauma, Peterborough City Hospital, Peterborough, GBR; 5 Plastic and Reconstructive Surgery, East and North Hertfordshire NHS Trust, Stevenage, GBR

**Keywords:** hand surgery, hand infection, flexor sheath washout, kanavel cardinal signs, flexor sheath infections

## Abstract

Flexor sheath infections (FSIs) are soft tissue infections affecting the hand, which, if mismanaged, can have devastating consequences. Clinical assessment is key to diagnosis, with many relying on Kanavel cardinal signs as an aid. To prevent unnecessary operative intervention and the associated post-operative combined patient and healthcare burden, it is key that patients with FSIs are correctly identified. It would also be useful to stratify severity of FSIs without surgical exploration. To date, there is no accepted method to assist clinicians in doing so. We retrospectively analysed data from a five-year period to see if we could identify pre-operatively (a) accurate predictors of FSIs and (b) severity of the FSIs. We established that only the presence of all four Kanavel cardinal signs significantly predicted the presence of an FSI. No other variable that was available prior to surgery could predict either presence or severity of infection.

## Introduction

Flexor sheath infections (FSIs) are closed space infections of the flexor tendon sheath in the hand. They can cause stiffness and, in some cases if left untreated, lead to impaired function or potentially require amputation [1-3]. Prompt diagnosis with early surgical management and antibiotic use are key elements to improve patient outcome [4-5]. Diagnosis of this infection is still heavily reliant on Kanavel cardinal signs (KCSs), initially proposed in 1912 [6].

KCSs are tenderness along the sheath (limited to the sheath), finger in fixed flexion, pain on passive extension and fusiform swelling of the digit.

The absence of one or more cardinal signs cannot exclude FSIs [1]. Despite being shown to have high sensitivity (91-97%), the specificity of the KCS is relatively low (51-69%) [7]. FSIs can often be mimicked by other types of hand infections or inflammatory conditions which require different management [2].

Clinicians increasingly rely on biochemical and physiological markers to support clinical history taking and examination to make an accurate diagnosis. Systemic signs of infection can be absent in FSIs, with fever being reported in only 17% of cases [2]. Blood tests for inflammatory markers (white blood cells [WBCs], C-reactive protein [CRP]) have low negative predictive values and therefore cannot be used to rule out FSIs [8].

This study analyses the experience of diagnosing and managing patients with suspected FSIs at a single centre in the United Kingdom. Our aim was to collect data available to the diagnosing clinicians, along with clinical findings recorded in theatre, to determine if we could establish any patterns or trends to help predict the diagnosis and perhaps even severity of FSIs.

## Materials and methods

Our plastic surgery unit is based at a district general hospital serving a catchment population of approximately 600,000. The department receives referrals from both the local emergency department and nearby district general hospitals, in addition to minor injury units and general practitioner (GP) surgeries. The mainstay of our FSI management involves admission, hand elevation, intravenous antibiotics and immediate flexor sheath washout under either regional or general anaesthesia.

Our preferred surgical technique used is the ‘minimal access washout’, similar to that described by Neviaser [9]. A small incision is made over the A1 pulley, and a further incision is made over the A5 pulley. An 18-gauge cannula is then inserted proximally, and the sheath is irrigated with normal saline. We do not employ post-operative irrigation of the sheath. This is converted to an open washout if deemed clinically necessary by the operating surgeon.

A retrospective analysis was undertaken of patient records who had undergone a surgical procedure to treat a suspected FSI.

Patients were identified through the hospital’s Clinical Information and Patient Tracking System (CIPTS). A search was performed on the ‘actual treatment’ field of the database with the following search terms: ‘flexor sheath washout’, ‘washout of flexor tenosynovitis’ and ‘washout of flexor tendon sheath’ for the five-year period between December 31, 2014, and December 31, 2019.

After exclusion of erroneously coded and unrelated procedures, 118 operations relating to washout of a suspected FSI were identified.

**Figure 1:**
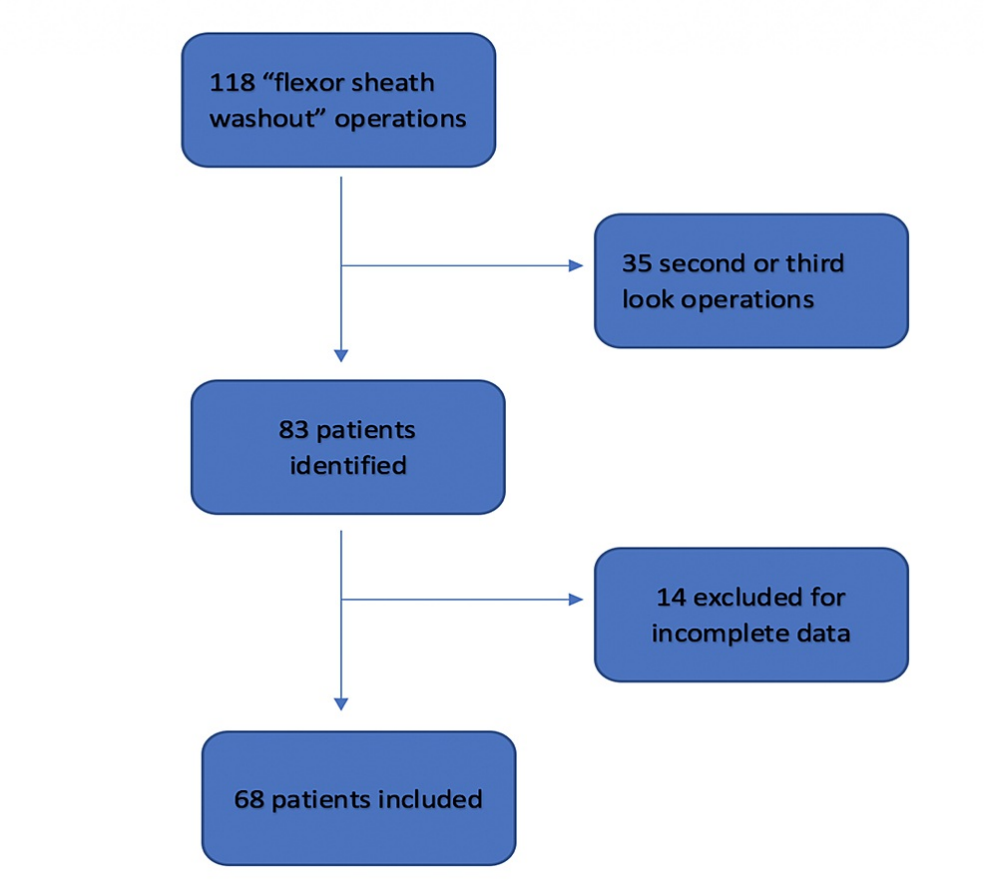
Flow diagram demonstrating data included and excluded from analysis

Physical patient records were retrieved from medical records. Notes pertaining to initial assessment and operative intervention were identified and analysed by authors. A total of 35 of the procedures were second- or third-look procedures and were excluded from further analysis. Records of 15 patients were excluded as they were incomplete (one had no intraoperative findings, three had no blood, 11 had no date of injury recorded). This yielded a total of 68 patients with primary washout procedures (Figure 1).

Notes were analysed and the following data were collected into a spreadsheet, as shown in Table 1.

**Table 1:** Data collected for each patient KCS, Kanavel cardinal signs; WBC, white blood cells; CRP, C-reactive protein

Data collected for each patient
Hospital number
Gender
Date of birth
Date of admission
Age on admission (years)
Date of injury
Timing of admission from injury (days)
Clinical findings on admission
Presence of KCS based on documented clinical findings
WBC (10^9^/L)
CRP (mg/L)
Intraoperative findings
Michon stage based on documented intraoperative finding

KCSs were calculated as a score ranging from 0 to 4 (we termed this the ‘Kanavel score’) based on documented clinical findings consistent with each of the cardinal signs. If the assessing clinician did not document any findings consistent with KCS, a score of 0 was awarded, and if findings consistent with all KCSs were documented, a score of 4 was awarded.

The intraoperative findings were used to retrospectively determine the Michon stage [10] and given a numerical value (0-normal findings, 1-turbid fluid, 2-pus in the sheath, 3-necrosis of sheath/tendon). This was then used to categorise the results into three groups: Michon 0, Michon 1 and Michon 2+. This broadly divided the patients into three groups: those who did not have intraoperative evidence of an FSI, those with a mild FSI and those with a severe FSI. The authors have chosen to use Michon 0 as a category. Michon did not include this category in his original staging system; we denote Michon 0 to those patients in whom there was no intraoperative findings suggestive of an FSI.

Both the Kanavel score and Michon stage were independently determined by authors J.M. and R.M., and any disagreements were resolved by discussion.

The authors’ standard for defining an FSI was that of a patient who had evidence of active infection within the sheath. This included any patient with intraoperative findings consistent with a Michon classification of 1 or more.

We initially compared groups to identify any statistically significant differences in age, time from injury, Kanavel score, WBC and CRP. We then utilised multivariate and ordinal logistic regression to determine if various data available to the diagnosing clinician would have been able to assist them in determining the likelihood of an FSI and, one step further, to postulate the severity of such an infection.

## Results

Of the 68 patients included, 39 were male and 29 were female. Fourteen patients were categorised as Michon 0, 20 as Michon 1 and 34 as Michon 2+.

Age and timing of injury from admission

The median ages and timing of injury to admission are shown in Table 2. The Kruskal-Wallis test demonstrated no statistical significance between the ages of the groups (p = 0.8196) or time from injury (p = 0.8568).

**Table 2:** Median values of the age and time from injury to admission of the Michon groups

	Median age (years)	Median time from injury to admission (days)
M0	50	1
M1	45.5	3
M2+	51	2

Kanavel score

We defined the Kanavel score as the number of KCSs present when examined. We identified a significant difference between Kanavel scores in each of the ‘Michon groups’ (p = 0.0004, Kruskal-Wallis test).

A key aim of this study was to establish whether it is possible to predict the presence of an FSI using pre-operative findings available to the assessing clinician in the emergency department setting. We utilised multivariate logistic regression techniques to establish which variables, including combinations of variables, could be used to calculate the probability of the presence of an FSI. The analysis demonstrated that the only variable able to predict the probability of a patient having an FSI is the Kanavel score. The authors define the presence of a true FSI based on intraoperative findings. This would include any patient with a Michon stage 1 or higher, which would suggest an active infection within the flexor sheath.

A further aim was to establish a method for predicting the severity of an FSI from findings available at the time of diagnosis. We used ordinal logistic regression to determine to what extent individual Kanavel scores were predictors of the probability of a patient having an FSI (Michon score of 1 or more). Our results demonstrated that only a Kanavel score of 4 was statistically significant for predicting the presence of FSI (Michon of 1 or more) (p=0.02). Table 3 displays the results odds of a patient having a particular Michon score with a pre-operative Kanavel score of 4. The reader will note odds of nearly 40 for the patient having a Michon score of 2 or 3, moderate-to-severe intraoperative findings and a pre-operative Kanavel score of 4.

**Table 3:** Odds of a patient having each Michon score intraoperatively with a pre-operative Kanavel score of 4

Michon score	Odds	95% CI
0 or 1	0.09	0.01-0.58
1 or 2	0.47	0.07-3.05
2 or 3	39.9	6.24-255.73

## Discussion

Whilst it is useful to confirm the value of the presence of all KCSs, it is disappointing that we could not find evidence from our dataset that the other scores and pre-operative data had a useful predictive value. This does highlight that making the diagnosis of FSI remains based on clinical assessment alone.

Unfortunately, this may lead to some patients unnecessarily undergoing a flexor sheath washout procedure in theatre. From our data, 14 patients who underwent such a surgery had intraoperative findings not consistent with an established FSI. Similar situations are reported by others in the literature and have prompted suggestions for alternative treatment of some cases of FSIs [11-12]. One could argue that intravenous antibiotics, in addition to hand elevation and close observation, could still have been appropriate in these patients, avoiding the potential risks of surgical intervention.

Giladi et al.’s review demonstrated improved outcomes associated with surgical techniques that employed limited entry into the flexor sheath and highlight that some of the complications of FSIs, including finger stiffness and tendon adhesions are potentially caused by aggressive surgical intervention. They further suggest that any surgical intervention is a potential influence on poor outcomes [5]. It seems logical to assume that any violation of the flexor sheath may induce scarring and naturally it would be better to avoid this where not required. The original management of early flexor sheath washout stems from a pre-antibiotic era but is mainstay and focus of FSI treatment to this day. This view does appear to be changing, with greater varying opinions on the management for this condition [12]. It is well known that antibiotic use significantly improves the outcomes for FSI patients [5], and several groups have demonstrated through case series successful management of FSI patients with antibiotics alone, negating the need for surgery altogether in most cases [3,11,12]. This alternative treatment does require a method to help risk-stratify patients to decide who requires surgery and who can be trialled with conservative methods.

There is no consensus on how to risk-stratify such patients at point of diagnosis [5]. It is therefore unclear for whom a conservative approach is appropriate. One group's approach was that those presenting early, without fluctuance and fewer than three KCSs would receive a trial of conservative management [13]. Our data demonstrate no statistically significant relationship between the timing of presentation and severity. We have also demonstrated that although the KCSs are useful, only a maximum score can be accurately used to predict severity.

Although microbiology cultures play a role in rationalising anti-microbial treatment, it is clear from other studies that pre-administration of antibiotics prior to sample collection can lead to questionable growths and sensitivities [14-15]. The impact of antibiotics prior to collection of samples for microbiology can be seen in our own data. Of 34 patients whose intraoperative findings were classed as 2 or 3 by the Michon classification (i.e. pus or purulent fluid present or worse), 12 (35%) patients’ microbiology was reported as ‘no growth’. This casts some doubt on the ability to use microbiological growth as the absolute diagnostic test to confirm an FSI.

Additionally, the use of microbiological information in diagnosis or risk stratification prior to surgery is not currently possible, as to obtain a result requires a surgical procedure to obtain the sample, in addition to time delays introduced by waiting for the culture result. The authors therefore did not include microbiology results in the study, as the results could not influence early management.

When considering other investigations available to the clinician for guiding FSI management, we found reports of success in diagnosing FSIs with point-of-care ultrasound (POCUS) in the emergency department [16-19]. Although promising, these reports only involve a small number of patients. Further studies are required to properly assess the reliability and validity of POCUS to aid pre-operative diagnosis of FSIs. It could be a useful adjunct in determining established FSI infection and could be used to stratify severity. One group reported a small case series on the use of limited flexor sheath washouts in the emergency department as an intervention used both to confirm purulence in the sheath and as a definitive treatment in some of their patients. Those who underwent a washout in the emergency department reported good functional outcomes [20]. Their study raises an interesting point: is there a role for limited incision or aspiration of the flexor sheath within the emergency department setting? This would help confirm the presence or absence of pus and therefore help guide further management. In addition, if this sample was taken prior to starting antibiotics, it would help yield more sensitive culture results, given the common practice of starting immediate broad-spectrum pre-operative antimicrobial treatment. There have been multiple advances in improving the timing and efficiency of antimicrobial susceptibility testing, including point-of-care antimicrobial testing [21]. If early antimicrobial susceptibility was identified in FSIs, it would certainly help target antibiotic usage early in these patients. Future research in these areas could prove to be interesting.

Patient risk factors for FSI severity have been identified [2]; the non-specific clinical signs and inflammatory markers pose a problem in determining severity. The ability to predict the severity of an FSI would be extremely valuable. It would guide the need and timing of surgery, improving outcomes for patients and potentially relieving pressure on already busy health services. We need more information to help us determine the severity of an infection.

Limitations of study

The authors recognise the limitations to the study design. Data were collected retrospectively and only included patients who were managed operatively. The study population was therefore prone to selection bias. Kanavel’s score and Michon’s stage were retrospectively determined from clinical findings and intraoperative findings respectively and prone to misclassification bias. In addition, it is difficult to retrospectively determine the inter-rater reliability for both the Kanavel score and intraoperative findings.

We also recognise that a larger data sample may increase the accuracy of our results and may reveal trends that we have not been able to detect. A prospective study with specifically defined criteria for determining the Kanavel score and the intraoperative Michon stage would help improve the inter-rater reliability. We hope that by highlighting the issues with the diagnosis of FSIs and the importance of establishing a method for predicting severity of infection, we might collaborate with other units to perform a multicentre data analysis.

## Conclusions

These results demonstrate that from our data set that the presence of all four KCSs was the only statistically significant predictor of the presence of an FSI. We have also demonstrated that the presence of all four KCSs, as expected, is linked to a near 40-fold chance of having a severe FSI (Michon score of 2 or 3). In addition to this, the results also highlight that a Kanavel score of 4 has a 95.3% positive predictive value for predicting findings consistent with FSIs.
